# Oral Pemphigoid Recalcitrant Lesion Treated with PRGF Infiltration. Case Report

**DOI:** 10.3390/dj9110137

**Published:** 2021-11-19

**Authors:** Eduardo Anitua, Mohammad H. Alkhraisat, Asier Eguia, Laura Piñas

**Affiliations:** 1 Clínica Eduardo Anitua, 01007 Vitoria, Spain; asier.eguia@ehu.eus (A.E.); lapica77@gmail.com (L.P.); 2 BTI Biotechnology Institute, 01007 Vitoria, Spain; mohammad.hamdan@bti-implant.es; 3 Department of Estomatology III, Faculty of Medicine and Nursing, University of the Basque Country, 48940 Leioa, Spain

**Keywords:** benign mucous membrane pemphigoid, autoimmune disease, vesiculobullous dermatoses, platelet rich plasma, case report

## Abstract

Mucous membrane pemphigoid (MMP) is a heterogeneous group of chronic autoimmune subepithelial blistering diseases. Oral involvement is present in almost all patients, may represent the onset of the disease, and causes different degrees of pain, dysphagia, soreness, and bleeding. Treatment is based on systemic and/or oral corticoids, or other immunosuppressants. Occasionally, oral lesions can show a poor response to standard treatments. We present the case of a 61-year-old female patient with a painful extensive MMP oral ulcerative lesion recalcitrant to previous systemic azathioprine and local triamcinolone treatment, which was successfully treated in a novel way using PRGF infiltrations as adjuvant. After four weekly infiltrations, pain was reduced from 10 to 0 in a VAS and the lesion was completely healed. The patient continued with a low dose maintenance immunosuppressive treatment (prednisone 5 mg/day PO), and after 13 months of follow-up, there was no relapse of the lesion and no side effects. Although future research is necessary to confirm these observations, PRGF could be a useful adjuvant for the management of extensive mucous membrane pemphigoid oral lesions.

## 1. Introduction

Mucous membrane pemphigoid (MMP) is a heterogeneous group of rare, chronic autoimmune and blistering diseases affecting mucous membranes and/or skin [1]. The onset of MMP is usually in the fifth to sixth decades and, as in other autoimmune diseases, it is more common in females (2–6:1) [2,3,4]. There is no known geographic incidence, and the precipitating event of the disease is unknown in most cases, although some cases can be drug-induced (by some antimicrobials, diuretics, anti-hypertensives, or NSAIDS), radiation induced (UV light or X-irradiation), or are related to concomitant malignancy (solid cancer or lymphoma) [1,2,3]. A genetic predisposition has also been suggested by an HLA-DQB1*0301 allele association [2].

In contrast to other vesiculobullous disorders as pemphigus or erythema multiforme, the autoantibodies in MMP are directed against different epithelial membrane zone (BMZ) proteins: BPAG2 (previously called BP180) or Collagen alpha-1(XVII) chain 180 kDa, BPAG1 (dystonin), integrin β4 subunit, and laminin 332 (formerly called laminin 5) [1,2,3]. Different pemphigoid subsets and clinical courses are determined by the specific targets of the autoantibodies in each patient. The genetic background and other environmental factors could also have a great influence in the diverse clinical pictures of this disease [1,2,3].

After a precipitating event that is unclear in most cases, IgG or IgA autoantibodies bind to the BMZ antigens, thereby activating a complement mediated inflammatory response in the subepithelial tissue, which provokes cytokine and leukocyte enzyme release. This leads to the detachment of the basal cells from the BMZ and the subsequent subepithelial blisters formation [5,6].

Most patients have oral involvement only, but skin, genital, ocular, laryngeal, or nasal lesions can be present. Patients with extraoral manifestations are considered “high risk” patients and may require more aggressive treatment [7]. Oral lesions are frequently the first manifestation and precede other systemic manifestations [8]. The diagnosis is based on clinical findings, histological and immunostaining examination, and immunoserology [1,8]. Early diagnosis and initiation of therapy influences the prognosis of the disease [1,2,3].

The most common sites for oral lesions are the palate and the gingiva. Desquamative gingivitis (DG) is the most frequent oral finding, and the sole involvement of the gingiva is not uncommon. Sometimes, it can be difficult to differentiate from the lichen or pemphigus related DG [8,9]. Blisters can remain for days, but normally are easily broken due to the trauma of eating or speaking, leaving persistent and painful irregular erosions and ulcers that are typically covered by a yellowish fibrinous membrane [8,9,10]. Pressure on a blister causes it to spread, which is known as Nikolsky’s sign. Vesicles or bullae are not commonly observed, as they burst very quickly [1,2,3]. Oral lesions have a chronic course and occasionally are recalcitrant to treatment [1,2,3]. Blisters quickly turn into painful ulcers, frequently presenting secondary infection and causing a remarkable impairment in the quality of life of the patients [8,9,10].

Multidisciplinary treatment is indicated for MMP patients, especially for “high risk” patients. Systemic corticoids alone or in conjunction with other immunosuppressive drugs are frequently necessary during a long time period to control the disease course. This prolonged treatment puts these patients in an increased risk of severe side effects [11]. Oral lesions also require prolonged treatment with different potency corticoids and other immunosuppressants [12].

MMP oral lesions that do not respond to immunosuppressive treatments remain a challenge for the clinician [13]. Although reported in some cases, the cause remains unclear, and there is still no standard treatment protocol accepted to manage this clinical situation [11,12,13]. In this work, we present the treatment and 13-months follow-up of a patient with MMP oral ulcers recalcitrant to systemic immunosuppressive therapy treated by the infiltration of plasma rich in growth factors (PRGF) as an adjunctive for ulcer healing.

## 2. Case Presentation

**Figure 1:**
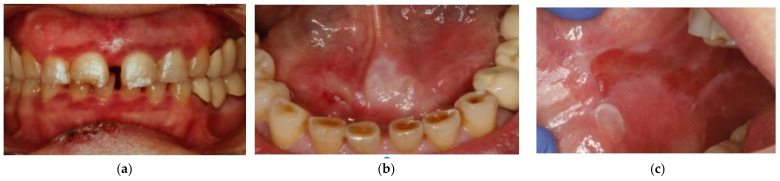
Initial state of the patient before starting treatment. The patient presented oral lesions localized in the (**a**) gingiva; (**b**) the floor of the mouth; (**c**) the right buccal mucosa.

A 61-year-old female patient presented in the clinic complaining of severe oral pain. The patient had a previous diagnosis of MMP and was on treatment with systemic azathioprine (100 mg/day) and artificial tears for chronic keratoconjunctivitis, and topical corticoids (triamcinolone acetonide 0.5% in aqueous solution, twice a day for two months) for oral lesions. The patient presented oral lesions localized in the gingiva, the floor of the mouth, and the right buccal mucosa (Figure 1).

After topical corticoids application, the lesions located in gingiva and in the floor of the mouth were resolved. However, the lesion located in the right buccal mucosa did not respond to the treatment and caused severe pain. For this reason, the patient requested adjuvant alternative treatment. She was then informed about the treatment protocol using perilesional infiltrations of plasma rich in growth factors (PRGF), and she gave her signed informed consent.

**Figure 2:**
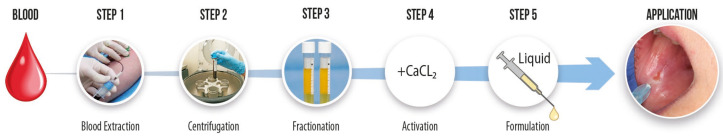
PRGF preparation protocol for the treatment of MMP oral lesions.

For the preparation of autologous plasma rich in growth factors (PRGF), an Endoret^®^-PRGF kit was used (KMU15, BTI Biotechnology Institute, Vitoria, Spain). Eighteen milliliters of the patient’s own blood were processed according to the manufacturer’s instructions (Figure 2). The volume of plasma obtained was fractionated into fraction-2 (F2), defined as the first 2 mL of plasma just above the buffy coat, and fraction-1 (F1), defined as the plasma volume above the F2. This allowed us to prepare a total volume of 4 mL of F2 plasma. Platelet activation was performed by adding 20 µL of 10% calcium chloride to each mL of F2. To facilitate the infiltration, activated F2 was loaded in a luer-lock syringe connected to a hypodermic needle 31G × 1/6. The PRGF was injected while still in liquid form. The infiltration was performed at four points surrounding the lesion, one time per week, and without previous local anesthesia. Pain was assessed using a visual analogical (VAS) score (in baseline and 7 days after each treatment), and clinical images were documented.

**Figure 3:**
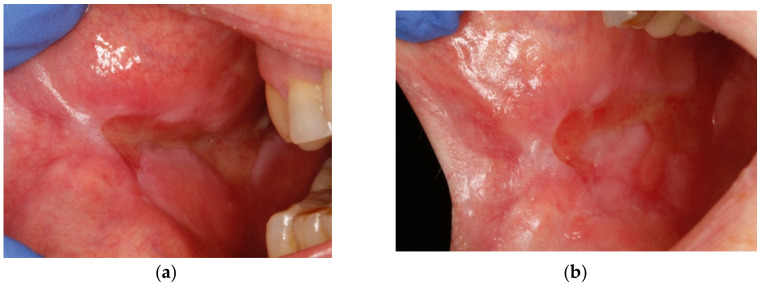

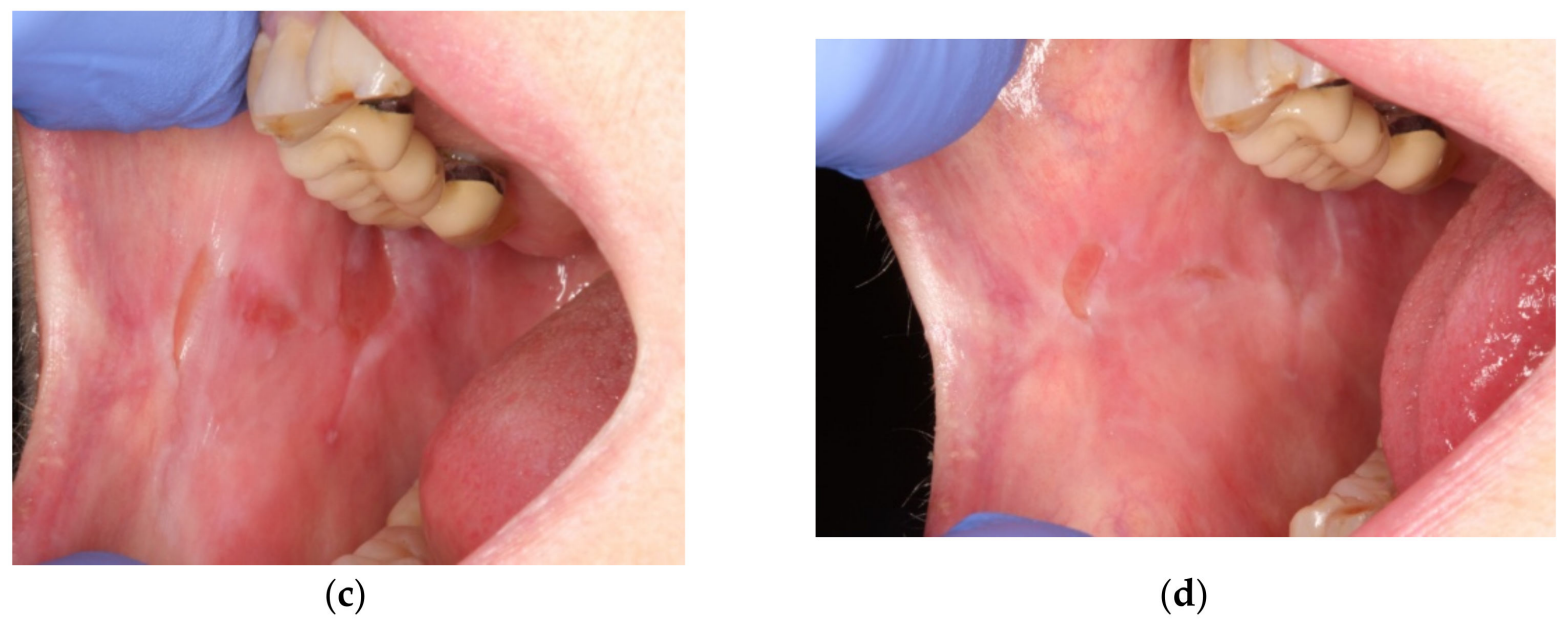
Clinical evolution. (**a**) The lesion after 7 days of the first infiltration. The size of the lesion was slightly reduced. (**b**) Image of the lesion 7 days after the second infiltration. The lesion was more superficial and less painful. (**c**) Seven days after the third infiltration. The lesion was almost resolved. (**d**) One week later. The lesion was resolved at 90%. At this point, we did no more infiltrations.

The perilesional infiltrations were carried out weekly, during 4 consecutive weeks Before the first one, the pain intensity described by the patient was of 10/10 points on the VAS scale. One week after the first infiltration, the pain intensity was reduced to 7/10 points, and the extension of the ulcerated lesion was reduced as well (Figure 3a). One week after the second infiltration, the pain intensity was reduced to 3/10, and the ulcerated lesion became less deep and presented a remarkable decrease in its extension (Figure 3b). After the third infiltration, the pain was completely resolved (0/10), and the extension of the lesion was further reduced (Figure 3c). Another week after the fourth infiltration (and the last one), the patient was free of pain and the lesion was almost totally healed (Figure 3d).

**Figure 4:**
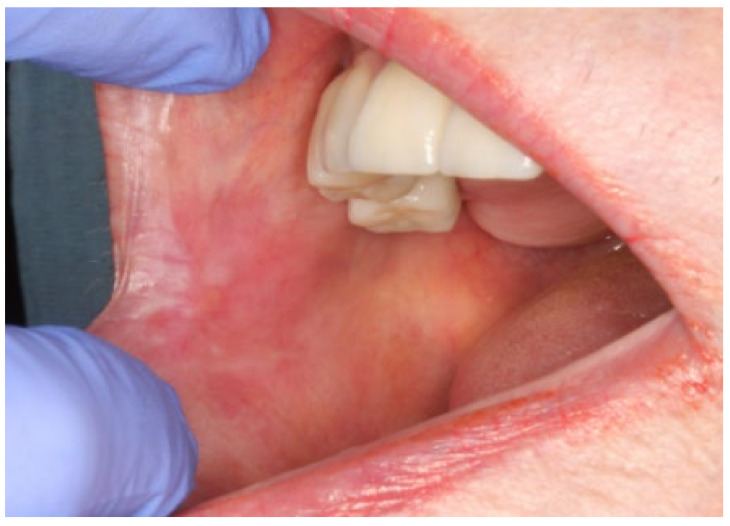
Two weeks after the last infiltration: complete resolution of the lesion.

**Figure 5:**
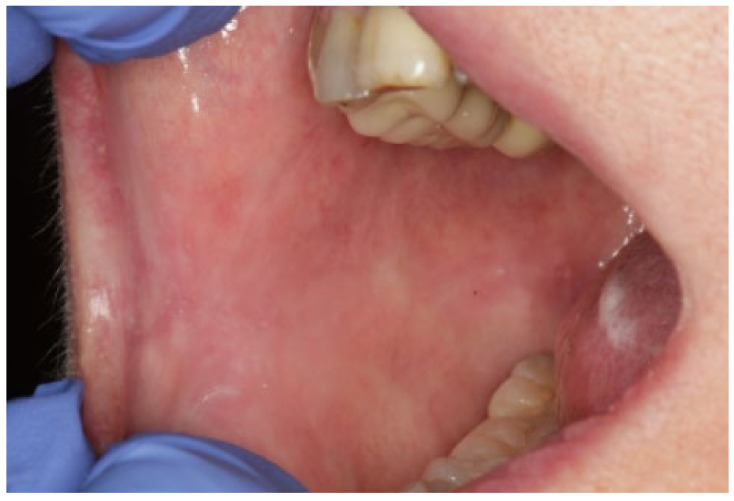
No relapse one year after the last infiltration.

**Figure 6:**
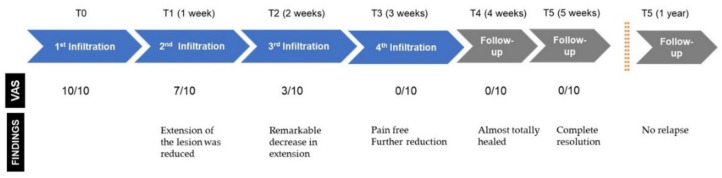
Timeline of the treatment and main outcomes.

The patient continued under periodic follow-up. Two weeks after the last infiltration, the lesion was completely healed (Figure 4). The patient was free of relapse during the 13 months after the first infiltration of PRGF and continued with systemic maintenance treatment (prednisone 5 mg/day PO). (Figure 5). A timeline and outcomes summary are presented in Figure 6.

## 3. Discussion

Different types of platelet-rich plasma (PRP) have been successfully employed to boost regeneration in several surgical procedures and clinical treatments in different fields of the medicine. Amongst many other applications, PRPs are currently widely used in wound healing, corneal ulcers treatment, chemical burns, or post-extraction dental socket regeneration [8]. The effects of PRP are based on the in situ liberation of a pool of biologically active proteins that influence and promote a range of biological processes, among them enhancing and accelerating the healing response [14].

Although several different protocols for PRPs are used in modern medicine, in this study, we employed PRGF, a totally autologous leukocyte free plasma, to prevent possible increases in IL-6, IL-1β, TNF-α, cyclooxygenase-2 (COX-2), nitric oxide, or synthase (iNOS) expression with the presence of high levels of leukocytes, which could interfere in pain relief [14,15].

The favorable effects observed in this case may have been promoted by the activity of different proteins present in PRGF such as the platelet-derived growth factor (PDGF), the transforming growth factor beta (TGF-β), the epithelial growth factor (EGF), fibronectin, vascular endothelial growth factor (VEGF), or fibroblast growth factor (FGF). They contribute to angiogenesis, collagen synthesis, endothelial cell migration and proliferation, or keratinocyte cell migration, proliferation, differentiation, growth, and migration, which are phenomena that are essential for healing and re-epithelialization [14,15].

On the other hand, different studies have supported the anti-inflammatory effect of PRGF [15,16]. This effect could be mediated by an inhibition of the transcription of nuclear factor kappa B (NF-κB) and the expression of COX2 and CXCR4 (chemokine receptor type 4), produced by the high content of hepatocyte growth factor (HGF) [17]. Recent studies have also demonstrated that PRGF reduces inflammatory markers expression such as intercellular adhesion molecule-1 (ICAM-1) and cyclooxygenase-2 (COX-2) expression in ocular surface cells cultured in a pro-inflammatory environment (fibroblasts treated with IL-1β and TNFα) [18]. Interestingly, IL-1β, TNFα, and other proinflammatory cytokines expression is impaired in pemphigoid patients, and even polymorphisms in these proinflammatory cytokine genes have been stated to have a possible role in the etiology of pemphigoids [19].

The development of an efficient three-dimensional (3D) fibrin scaffold formation that occurs after PRGF administration could also have specific positive effects for healing, helping some cell populations to guide their position and function [14,15].

Previous experience with the use of PRPs as adjuvant treatment in oral lesions of mucocutaneous diseases is still scarce. El-Komy et al. [20]. reported good results in pain relief and lesions healing in 6/7 patients with oral pemphigus vulgaris lesions recalcitrant to standard treatments. PRGF has been employed in one recent study [21] to treat four patients with recalcitrant ulcerative oral lichen planus (OLP) lesions, following the same protocol employed at the present study. The results of the treatment of these OLP patients were completely satisfactory, with a complete healing of the ulcerative recalcitrant lesions.

## 4. Conclusions

To the best of our knowledge, this is the first case report in the literature of recalcitrant MMP oral ulcerative lesions successfully treated with PRGF. The protocol employed allowed for rapid pain relief and a good clinical response, without side effects or relapse of the lesion during one year.

PRGF could be a useful adjuvant therapy for the management of oral lesions of mucous membrane pemphigoid. Future research is necessary to confirm the outcomes of these observations.

## Data Availability

All the data obtained in this research are described in the manuscript.
